# The Termination of Honorary Staff Appointments

**Published:** 1916-01-29

**Authors:** 


### The Hospital

The Workers' Newspaper of Administrative Medicine and Institutional Life, Administration, National Insurance and Health.

No. 1546, Vol. LIX. SATURDAY, JANUARY 29, 1916. PRICE ONE PENNY.

### THE TERMINATION OF HONORARY STAFF APPOINTMENTS.

There are, broadly speaking, three systems of appointment of the visiting medical staff at voluntary hospitals. One is the appointment of such officers as a permanency, with or without an age limit, on an honorary basis. Another is to make the appointments for stated periods, usually of five years, with eligibility for re-election. The third is to grant a small annual honorarium, which makes the appointment terminable by either side on twelve months' notice, under the usual rule governing the relations of employers and employees. Under the last system each party to the contract knows the position, and the only possible question that can arise is whether the institution has the right to enforce instant dismissal on payment of a year's honorarium in lieu of notice. The disadvantage of the second system is that it might become an arguable proposition whether the institution has the right of dismissal at all, on the ground that the quinquennial re-election is the correct way of getting rid of an unsatisfactory officer. We do not think such a position is tenable, but it might be advanced with some plausibility if a case were ever taken to the Courts.

The first system is that which is still most usual, though a good many hospitals have abandoned it in recent years in favour of one or other of the alternatives. In some cases there is definite provision in the statutes for the dismissal of members of the honorary staff, with conditions as to a prescribed majority of the board of governors necessary to give its decision validity. Often there is no such provision, so that an element of doubt creeps in as to exactly what rights the managers and their professional staff respectively possess. Fortunately cases in point are rare, but they do occur. There was one not very long ago at one of the best-known voluntary hospitals in London; and quite recently, we learn, there has been one at another institution of high standing. The circumstances of this last example, so far as we can gather them, make the case of some interest to hospital managers as a matter of principle. Seeing they might conceivably govern some future case, we think it right briefly to describe them.

A member of the profession was elected some years ago to the honorary visiting staff of a hospital which had no provision in its laws (except an age limit) for the termination of the appointment. The temperament and disposition of this practitioner turned out to be such that at recurring intervals he quarrelled with those with whom he had to work: with his medical colleagues of the staff, with resident medical officers, with members of the nursing staff from its head downwards, with male employees, and with the governing board. After much and increasing friction it seemed good to the board (without a dissentient, we believe) to dismiss this officer from his appointment. Counsel's opinion was taken, and the board was advised that they had a right to dispense at once with the doctor's services, but that a year's notice might place them in a stronger position if it came to legal proceedings. On this advice the doctor was informed that his resignation would be accepted, and that if it was not sent in, his appointment would be terminated in twelve months' time. The medical man, it is said, had himself taken counsel's opinion, and had announced that he was prepared to fight any such sentence of dismissal. However, in the end he decided not to do so, and sent in his resignation. Thus the incident terminated, and it may have value if it serves as a precedent which may be followed in the future by any other institution which experiences a difficulty in getting rid of an officer whose services are no longer desired. The position taken up by the board was, under the circumstances, in the best interests of the hospital. The regulations of every hospital ought to contain powers of dismissal.

